# Newly developed anti-angiogenic therapy in non-small cell lung cancer

**DOI:** 10.18632/oncotarget.23755

**Published:** 2017-12-26

**Authors:** Jingjing Qu, Yongchang Zhang, Xue Chen, Haiyan Yang, Chunhua Zhou, Nong Yang

**Affiliations:** ^1^ Department of Lung Cancer and Gastrointestinal Oncology Medicine, Hunan Cancer Hospital, Affiliated Cancer Hospital of Xiangya School of Medicine, Changsha, 410013, China

**Keywords:** angiogenesis, combined with chemotherapy, tyrosine kinase inhibitor, immunotherapy, NSCLC

## Abstract

Angiogenesis and its role in the growth and development of non-small cell lung cancer (NSCLC) metastases has become an increasing clinical problem. Vascular endothelial growth factor (VEGF) plays a key role in advanced NSCLC. To some extent, anti-angiogenic therapies acquired some efficacy in combination with chemotherapy, target therapy and immunotherapy. However, the reliable clinical benefit obtained with these drugs is still questionable and often quantitatively limited. In this review, the authors highlight the data obtained from first-line, second-line, epidermal growth factor receptor tyrosine kinase inhibitor(EGFR-TKI) target therapy and immunotherapy in NSCLC patients who are treated with anti-angiogenic molecules in advanced NSCLC. The purpose of this study is to help us truly understand how to best use angiogenesis therapy in advanced NSCLC.

## INTRODUCTION

Treatment strategies of lung cancers have expanded greatly in recent years with the development of targeting therapy in cancer-specific oncogenic driver mutations, such as the epidermal growth factor receptor (EGFR), the anaplastic lymphoma kinase (ALK) and ROS1 rearrangements. However, these molecularly targeted therapeutic strategies have been restricted to the development of secondary resistance, which has led to treatment failure.

Angiogenesis plays a major role in the development, progression and metastatic spread of solid tumors [1]. As far as we know, there are three main types of angiogenesis in physical or pathological situations. Under physiological circumstances, the development of the vasculature involves the birth of new endothelial cells and their assembly into tubes, in addition to the sprouting (angiogenesis) of new vessels from existing ones during embryogenesis. Following this morphogenesis, the normal vasculature becomes largely quiescent. Second, in the adult, as part of physiologic processes such as wound healing and female reproductive cycling, angiogenesis is turned on, but only transiently. During carcinogenesis, an “angiogenic switch” is almost always activated and remains on, causing the normally quiescent vasculature to continually sprout new vessels that help sustain expanding neoplastic tumor growths, maintain nutrients and oxygen and evacuate metabolic wastes and carbon dioxide [2]

**Figure 1:**
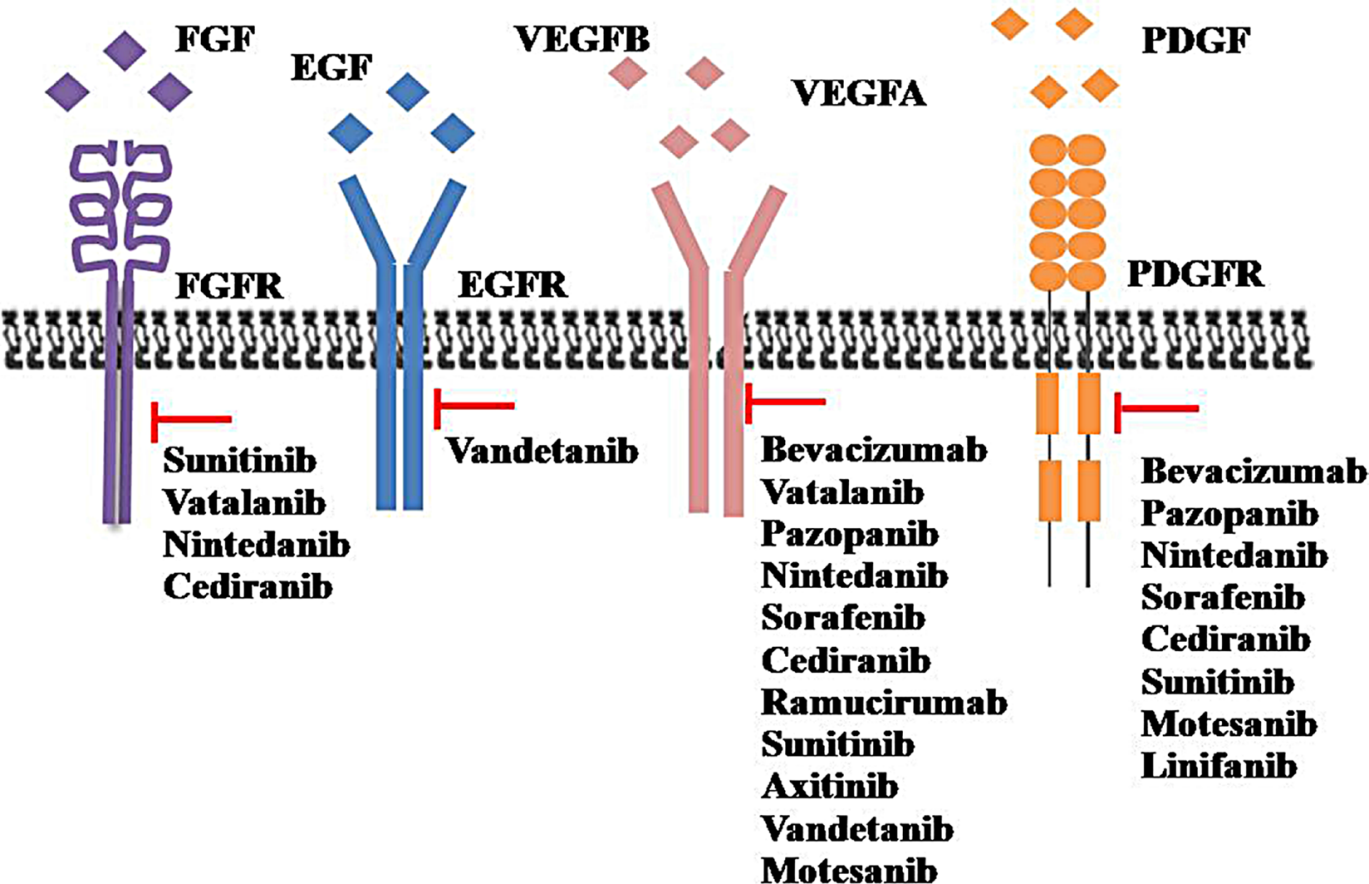
The main anti-angiogenic with their relevant potential targeted molecules FGF: Fibroblast growth factor; FGFR: Fibroblast growth factor receptors; EGF: Epidermal growth factor; EGFR: Epidermal growth factor receptor; VEGF: Vascular endothelial growth factor; VEGFR: Vascular endothelial growth factor receptor; PDGF: Platelet-derived growth factor; PDGFR: Platelet-derived growth factor receptor.

Several molecular drivers and signaling pathways are involved in tumor angiogenesis. The pro-angiogenic factors include vascular endothelial growth factor (VEGF), platelet-derived growth factor (PDGF), fibroblast growth factor (FGF), and the angiopoietins (Figure 1) [3]. The VEGF family includes VEGFA (usually referred to as VEGF), VEGFB, VEGFC, VEGFD, and platelet-derived growth factor (PDGF). VEGF stimulates angiogenesis via VEGF receptors (VEGFRs) and ligands. When ligands are activated, VEGFRs lead to dimerization and auto-phosphorylation and activates the downstream signaling pathway. This leads to endothelial cell survival, proliferation, and migration. VEGF also induces vasodilation and acts as a vascular permeability factor, which underlies its significance in tissue inflammation and the tumor microenvironment.

To establish the concrete benefit of the application of an anti-angiogenic strategy in lung cancer treatment, several clinical trials have been conducted or are currently ongoing. The aim of this review is to collect the main anti-angiogenic molecules derived from the first line, second line and target therapy trials conducted in non-small cell lung cancer (NSCLC) patients.

### Combination of antiangiogenic and chemotherapy in first-line of NSCLC

#### Bevacizumab

Bevacizumab is a monoclonal antibody that works directly against VEGF. This preliminary result prompted a phase III trial (ECOG 4599) conducted by the Eastern Cooperative Oncology group, which resulted in FDA approval of bevacizumab in combination with carboplatin and paclitaxel for patients with non-squamous NSCLC [4].In this study, the addition of bevacizumab to paclitaxel plus carboplatin in the treatment of NSCLC has a significant survival benefit. Another phase III trial (AVAiL) was conducted to evaluate the effect of bevacizumab in combination with gemcitabine and cisplatin in first-line management of advanced NSCLC [5]. The AVAiL trial confirms the efficacy of bevacizumab when combined with this chemotherapy regimen. To evaluate the safety and efficacy of first-line bevacizumab-contained chemotherapy in a broader patient population, the SAiL study recruited 2212 patients with advanced or recurrent non-squamous NSCLC from 40 countries across six continents. The results demonstrated that bevacizumab has manageable and acceptable safety as a first-line therapeutic combined with standard chemotherapy [6]. The results of the SAiL trial are further confirmed by another phase IV trial (ARIES), which conducted the analysis of several age subgroups in 1967 advanced non-squamous NSCLC patients. Progression-free-survival (PFSs) across each age subgroup were similar, while the overall survival (OS) in younger patients (≤ 65and ≤ 75 years) was better than older patients (≥ 65 and ≥ 75 years). This study indicated that bevacizumab in combination with chemotherapy is a viable first-line treatment option for the elderly with advanced non-squamous NSCLC [7]. A Japanese randomized phase II trial (JO19907) showed a significantly increased ORR but no improvement of median OS in patients with carboplatin-paclitaxel plus bevacizumab versus chemotherapy alone [8]. The clinically meaningful benefits of adding bevacizumab to carboplatin and paclitaxel in Chinese patients was further confirmed in a phase III BEYOND trial. Those positive results demonstrated the efficacy of bevacizumab in the Asian populations [9]. All the specific data are shown in Table 1. On the other hand, countless numbers of trials have been conducted with bevacizumab in NSCLC. The most recent data are reported in Table 1 [10–14].

**Table 1:** Combination of bevacizumab and chemotherapy in first-line of NSCLC

Agent	Target	Arms	No	Stage	ORR of (%)	PFS (ms)	OS (ms)	Note	Ref
Bevacizumab	VEGF	Paclitaxel +carboplatin Paclitaxel+carboplatin +bevacizumab	444 434	IIIB or IV		4.5 6.2	10.3 12.3	Excellent results with an excellent PFS and OS	[4]
		Cisplatin+ gemcitabine Cisplatin+ gemcitabine +7.5 mg/kg bevacizumab Cisplatin+ gemcitabine +15 mg/kgbevacizumab	347 345 351	Advanced NSCLC	20.1 34.1 30.4	6.1 6.7 6.5	-	The secondary end point of OS is currently immature because of limited follow-up	[5]
		Taxane-containing regimen Cisplatin doublets Bevacizumab were carboplatin doublets	794 829 1087	Advancednon-squamous NSCLC	-	7.8 7.6	- 14.3 14.7	Acceptable safety as a first-line combined with standard chemotherapy	[6]
		Bevacizumab with chemotherapy < 65 years > 65 years < 75 years > 75 years	954 1013 1597 370	Advanced non-squamous NSCLC		6.4 6.8 6.6 6.6	14.2 12.1 13.5 11.6	Bevacizumab in combination with chemotherapy is a viable first-line treatment option for elderly with advanced non-squamous NSCLC.	[7]
		Carboplatin-paclitaxel Bevacizumab+ carboplatin-paclitaxel	59 121	IIIB/IV non-squamous NSCLC	31.0 60.7	5.9 6.9	> 22ms > 22 ms	Not sufficiently powered to assess the OS benefit of bevacizumab plus CP	[8]
		Carboplatin-paclitaxel Bevacizumab+ carboplatin-paclitaxel	138 138	Advanced non-squamous NSCLC	26 54	6.5 9.2	17.7 28.5	Excellent results with an excellent PFS and OS in Chinese	[9]
		Bevacizumab+carboplatin-pemetrexed Brain metastases Without brain metastases	11 28	Advanced non-squamous NSCLC	-	8.2 8.0	14.0 12.0	The significant in brain metastases was unclear because of the smaller sample	[10]
		Paclitaxel/carboplatin + axitinib Paclitaxel/carboplatin + bevacizumab	58 60	IIIB/IV	29.3 43.3	5.7 6.1	10.6 13.3	Discontinued for lack of efficacy and safety issues	[11]
		Carboplatin/paclitaxel + bevacizumab	67	IV non-squamous NSCLC with asymptomatic brain metastases	62.7	6.7	16.0	Encouraging efficacy and acceptable safety with NSCLC and asymptomatic, untreated brain metastases	[12]
		Pemetrexed+ bevacizumab	12	III/IV Non-squamous NSCLC age ≥70	25	5.4	13.6	Well tolerated and shows promise as first-line treatment for elderly NSCLC patients	[13]
		Carboplatin- paclitaxel+ bevacizumab	36	III/IV Non-squamous NSCLC age ≥70	-	8.4	29.2	Feasible, effective first-line regimen for elderly NSCLC patients	[14]

#### Ombrabulin

Ombrabulin is a vascular disrupting agent (VDA) that stops the tumor blood flow independent of the tumor site and the specific type of cancer (Table 2) [15]. The DISRUPT trial showed that 176 patients with metastatic NSCLC received ombrabulin 35 mg/m^2^ or placebo followed by a taxane–platinum regimen every 3 weeks. The results demonstrate that the median PFS was not significantly improved with ombrabulin vs placebo (5.65 vs 5.45 months; *P* = 0.39). The two groups showed a similar OS (median 11.0 months in both groups), ORR (32% ombrabulin VS 31% placebo) and similar safety profiles [16].

**Table 2:** Combination of the other antiangiogenic and chemotherapy in first-line of NSCLC

Agent	Target	Arms	No	Stage	ORR of (%)	mPFS	mOS	Note	Ref
Axitinib	VEGF	Axitinib 5 mg orally	32	Advanced NSCLC.	9	4.9	14.8	Single-agent activity and well tolerated with manageable toxicities NSCLC.	[19]
		Paclitaxel/carboplatin + axitinib Paclitaxel/carboplatin + bevacizumab	58 60	IIIB/IV	29.3 43.3	5.7 6.1	10.6 13.3	Discontinued for lack of efficacy and safety issues	[11]
		Pemetrexed/cisplatin Pemetrexed/cisplatin + axitinib Continuously Pemetrexed/cisplatin + axitinib days 2 - 19	57 55 58	Advanced non-squamous NSCLC	26.3 45.5 39.7	7.1 8.0 7.9	15.9 17.0 14.6	No significant difference in terms of both PFS and OS	[20]
Ramucirumab	VEGF	Pemetrexed-platinum Ramucirumab+pemetrexed-platinum	71 69	Advanced/metastatic NSCLC	38.0 49.3	5.6 7.2	10.4 13.9	No significant difference in PFS,OS and ORR	[21]
		Paclitaxel/carboplatin + ramucirumab	40	Advanced NSCLC	55	7.8	16.8	The study resulted in a 6-month PFS rate and safety profile	[22]
Bavituximab	PS	paclitaxel-carboplatin+ Bavituximab	49	IIIB/IV NSCLC	40.8	6.0	12.4	demonstrated a tolerable safety profile and potential efficacy	[24]
Linifanib	VEGR PDGFR	Carboplatin- paclitaxel linifanib 7.5 mg+ Carboplatin- paclitaxel linifanib 12.5 mg+ Carboplatin- paclitaxel	47 44 47	IIIB/IV non-squamous NSCLC	25.5 43.2 31.9	5.4 8.3 7.3	11.3 11.4 13.0	Improved PFS with a modest trend for survival benefit	[26]
Cediranib	VEGR PDGFR FGFR	Gemcitabine/carboplatin Cediranib +Gemcitabine/carboplatin	29 58	IIIB/IV NSCLC	20.0 19.0	4.5 6.3	9.9 12.0	Did not meet its primary endpoint of ORR but met its secondary endpoint of PFS	[27]
		Carboplatin- paclitaxel Cediranib + Carboplatin- paclitaxel	144 143	Advanced NSCLC	-	5.5 5.5	12.1 12.2	Did not significantly improve PFS or OS	[28]
		Paclitaxel/carboplatin 30 mg cediranib +Paclitaxel/carboplatin	125 126	Advanced NSCLC	16.0 38.0	5.0 5.6	10.1 10.5	30 mg dose not tolerable for excessive toxicities	[29]
Pazopanib	VEGFR, PDGFR c-Kit	Pemetrexed- cisplatin Pemetrexed+ pazopanib	62 35	Advanced non-squamous NSCLC	34 23	22.9WS 25.0WS	-	Median OS could not be estimated based on the collected data before the study was closed and survival follow-up ceased	[31]
Motesanib	VEGFR, PDGFR c-Kit	Carboplatin/paclitaxel + motesanib 125 mg Carboplatin/paclitaxel + motesanib 75 mg twice daily Carboplatin/paclitaxel + bevacizumab	61 62 63	Advanced non-squamous NSCLC	30 23 37	7.7 5.8 8.3	14 12.8 14	The efficacy of 125 mg motesanib or bevacizumab was comparable	[33]
		Carboplatin/paclitaxel Carboplatin/paclitaxel + motesanib	549 541	IIIB/IV non-squamous NSCLC	26 43	5.4 5.6	11.0 13.0	No significantly improve OS	[34]
		Carboplatin/paclitaxel Carboplatin/paclitaxel+ motesanib 125 mg	178 182	IIIB/IV or recurrent squamous NSCLC	35 38	5.1 4.9	10.7 11.1	No significantly of OS ,PFS and had unacceptable toxicity	[35]
Vandetanib	VEGFR EGFR	Vandetanib Vandetanib+ Carboplatin/paclitaxel Carboplatin/paclitaxel	73 56 52	Advanced NSCLC	7 32 30	11.5 ws 24.0 ws 23.1 ws	10.2 10.2 12.6	Vandetanib monotherapy had shorter PFS	[36]
		Gemcitabine Gemcitabine + vandetanib	63 61	Advanced NSCLC	-	169 d 183 d	-	Significant prolongation of PFS	[38]

#### Axitinib

Axitinib is an oral, potent, selective inhibitor of VEGFR. Single agent axitinib reduced micro-vessel density and induced tumor necrosis in a murine Lewis lung carcinoma model and demonstrated dose-dependent inhibition of the tumor growth [17]. Axitinib demonstrated single-agent activity in patients with advanced NSCLC [18]. Single use axitinib demonstrated an anti-tumor response in two patients with NSCLC. A phase II study evaluated the efficacy and safety of single-agent axitinib. In this study, the median PFS was 4.9 months overall. The median OS was 14.8 months in patients receiving first-line axitinib [19]. A randomized Phase II of first line axitinib or bevacizumab combined with paclitaxel/ carboplatin therapy for patients with advanced NSCLC did not improve the efficacy and was not as well tolerated. The median PFS for axitinib and bevacizumab was 5.7 and 6.1 months, respectively [HR 1.09; 95% CI: 0.68–1.76; P = 0.64], while the median OS was 10.6 and 13.3 months (HR 1.12, 95% CI 0.74–1.69; *P* = 0.70). The ORRs were 29.3% and 43.3% [11]. Another randomized Phase II study recruited 170 patients. The patients received axitinib with or without combined pemetrexed and cisplatin that resulted in non-significant differences in PFS and OS [20]. However, further trails need to be adapted for clinical evaluation.

#### Ramucirumab

Ramucirumab is a monoclonal antibody that specifically targets the domain of VEFR receptor 2. A randomized first-line Phase II trial was conducted to compare the efficiency of pemetrexed and carboplatin (or cisplatin) and ramucirumab plus pemetrexed and carboplatin (or cisplatin) once every 3 weeks. No significant difference was seen in the PFS (5.6 months for the pemetrexed-platinum and 7.2 months for the ramucirumab-pemetrexed-platinum, *P* = 0.132) or the ORR (8.0% and 49.3% for the pemetrexed-platinum and ramucirumab-pemetrexed-platinum arms, respectively *P* =0 .180). However, there is a significant disease control rate between pemetrexed-platinum and the ramucirumab-pemetrexed-platinum (70.4% VS 85.5%, *P* = 0 .032). This study showed that ramucirumab has clinical activity in combination with pemetrexed and platinum in non-squamous NSCLC patients [21]. The other first-line Phase II study investigating whether the addition of ramucirumab improves the PFS in advanced NSCLC. Forty patients with advanced NSCLC received ramucirumab followed by paclitaxel and carboplatin on day 1 every 21 days as a first-line therapy. The 6-month PFS rate was 59.0%, and the ORR was 55.0%. This study validates that ramucirumab in combination with paclitaxel-carboplatin results in a 6-month PFS rate and safety profile [22].

#### Bavituximab

Bavituximab is a chimeric monoclonal antibody directed against the membrane phospholipid phosphatidylserine (PS). Bavituximab enhanced antitumor immunity. Several preclinical investigations have demonstrated the efficacy of bavituximab in combination with other modalities against the development of multiple cancers [23]. An open-label Phase II study was conducted to assay the efficiency of bavituximab in patients with stage IIIB/IV NSCLC. The 175 mg/m^2^ of paclitaxel was administered every 21 days with weekly treatments of 3 mg/kg bavituximab followed by bavituximab monotherapy were conducted in 49 patients with up to six cycles of carboplatin until progression or unacceptable toxicity. The primary efficacy endpoint of the ORR was 40.8%. The median PFS and OS were 6.0 and 12.4 months, respectively. This study showed bavituximab in combination with paclitaxel-carboplatin with a tolerable safety profile and potential efficacy in patients with advanced NSCLC [24].

#### Linifanib

Linifanib is a potent, orally active, and selective inhibitor of VEGR and PDGFR kinase activities with clinical efficacy in NSCLC. A phase I dose-escalation study evaluated the pharmacokinetics, safety, and efficacy of linifanib in combination with carboplatin/paclitaxel in Japanese patients with advanced NSCLC. The results showed that 12.5 mg linifanib added to carboplatin/paclitaxel is well tolerated in Japanese patients with advanced/metastatic NSCLC [25]. Recently, the results of another Phase II trial evaluating linifanib (7.5 mg VS 12.5 mg) with carboplatin and paclitaxel as first-line therapy of advanced non-squamous NSCLC were evaluated. Median PFS times were 5.4 months with chemotherapy alone, 8.3 months in linifanib 7.5 mg combine with chemotherapy and 7.3 months in linifanib 12.5 mg combine with chemotherapy. Median OS times were 11.3, 11.4, and 13.0 months in the chemotherapy, 7.5 mg linifanib, and 12.5 mg linifanib arms, respectively. Both linifanib doses were associated with increased toxicity, particularly related to VEGF/PDGF inhibition [26].

#### Cediranib

Cediranib (AZD2171) is an oral tyrosine kinase inhibitor of all three VEGFRs, PDGFR and FGFR that has shown antitumor activity, including NSCLC in phase I studies. A randomized phase II study was conducted to assay the safety and efficacy of gemcitabine and carboplatin with (arm A) or without (arm B) daily oral cediranib as a first-line therapy for advanced NSCLC. A total of 58 and 29 evaluable patients were accrued to arms A and B. The study met its secondary PFS end point (PFS in arm A was 48%, 95% CI: 35%–62%), thus meeting the protocol-specified threshold of at least 40%. The ORR was 19% and the VS was 20% (*p* = 1.0). The median OS was 12.0 versus 9.9 months (*p* = 0.10). Hence, the trial did not meet its primary ORR end point, but met its secondary PFS end point, resulting in increased toxicity [27]. A randomized double-blind trial of carboplatin and paclitaxel with daily oral cediranib or placebo in advanced NSCLC was performed. The data showed that the median OS was 12.2 and 12.1 months for cediranib and placebo, respectively (*p* = 0.72) and the median PFS was 5.5 months in each group (*p* = 0.49). However, the response rates (RRs) were 52% VS 34% in the cediranib and placebo arms (*p* = 0.001) with a median response duration of 4.3 (cediranib) and 4.2 months (placebo), which showed cediranib daily to carboplatin/paclitaxel chemotherapy increased RR, but not survival [28].

On the basis of a Phase II randomized trial, it was demonstrated that the addition of cediranib to carboplatin/paclitaxel in the first-line treatment of NSCLC resulted in improved PFS (5.6 vs 5 months; *p* = 0.13), but did not appear tolerable at a 30-mg dose. Maybe a randomized double-blind placebo-controlled trial of cediranib 20 mg with carboplatin and paclitaxel in advanced NSCLC will be initiated [29].

#### Pazopanib

Pazopanib is an orally active, small molecule inhibitor targeting multiple tyrosine kinases (VEGFR, PDGFR and c-Kit). A study was designed to evaluate pazopanib as a maintenance therapy after standard first-line chemotherapy in advanced NSCLC patients. The median OS was 17.4 months for pazopanib and 12.3 months for the placebo (*p* = 0.257). The median PFS was 4.3 months versus 3.2 months (*p* = 0.068). However, this study was stopped due to a lack of efficacy by stringent PFS criteria at a futility interim analysis [30]. The randomized open-label phase II study evaluated the efficacy, safety, and tolerability of pazopanib in combination with pemetrexed compared with the cisplatin/pemetrexed in first-line patients with previously untreated, advanced, non-squamous NSCLC. However, the PFS between the pazopanib/pemetrexed and cisplatin/pemetrexed arms were not significantly different (25.0 versus 22.9 weeks, respectively; *p* = 0.26) or ORR (23% versus 34%, respectively; p = 0.21). Meanwhile, the OS could not be estimated based on the data collected before the study was closed and survival follow-up ceased. Unfortunately, there were three unacceptable levels of toxicity in the pazopanib/pemetrexed arm, including the ileus, tumor embolism, and bronchopneumonia/sepsis due to the final study termination [31].

#### Motesanib

Motesanib is a small-molecule antagonist of VEGFR, PDGFR and c-Kit. The safe maximum tolerated dose (MTD), and pharmacokinetics of motesanib were explored in advanced NSCLC in a phase IB study. It showed that motesanib was tolerable when combined with carboplatin/paclitaxel with little effect on motesanib pharmacokinetics at the 125 mg once daily dose level. However, it needed further investigation [32]. A phase II study estimated the efficacy of paclitaxel–carboplatin (PC) plus motesanib in advanced non-squamous NSCLC. The ORR was 30%, 23%, and 37% in the carboplatin/paclitaxel and motesanib 125 mg (arms A) VS carboplatin/paclitaxel + motesanib 75 mg twice daily (arms B) and PC + bevacizumab (arms C). The median PFS in arm A was 7.7 months; for arm B, it was 5.8 months; and for arm C, it was 8.3 months. The median OS for arm A was 14.0 months; for arm B, it was 12.8 months; and for arm C, it was 14.0 months. Taken together, this study demonstrated that 125 mg motesanib or bevacizumab plus PC were recommended, but they had higher toxicity [33]. Another international, randomized placebo-controlled double-blind phase III study was conducted to analyze whether motesanib plus CP will improve the OS in advanced non-squamous NSCLC. The median PFS was 5.6 months VS and 5.4 months in the carboplatin/paclitaxel plus motesanib group compared with carboplatin/paclitaxel alone (*P* < 0.001). The ORR was 43% versus 26% (*P* < 0 .001). It is disappointing that the there was no significant difference in the median OS between the two groups (13.0 VS 113.0, *P* = 0.14) and in the adenocarcinoma subset [34]. The phase 3 MONET1 study evaluated motesanib plus carboplatin/paclitaxel (Arm A) versus placebo plus carboplatin/ paclitaxel (Arm B)as a first-line therapy for stage IIIB/IV or recurrent squamous NSCLC. The median OS time was 11.1 months compared with 10.7 months (Arm A VS Arm B, *p* = 0.3306). The median PFS times were 4.9 months and 5.1 months (Arm A VS Arm B, *p* = 0.2294). The ORR in Arm A was 38% compared with 35% in Arm B (*p* = 0.7362). Most importantly, the motesanib plus PC group showed more serious adverse events, especially bleeding events [35].

#### Vandetanib

Vandetanib is a once daily oral inhibitor of VEGFR and epidermal growth factor receptor(EGFR) signaling. A randomized Phase II Study of vandetanib alone or with paclitaxel–carboplatin (PC) as a first line therapy in advanced NSCLC showed that vandetanib plus PC decreased the risk of progression with longer PFS [36]. A Phase I study investigated the safety, pharmacokinetics and tolerability of vandetanib with either gemcitabine plus cisplatin (GC) or vinorelbine plus cisplatin (VC) in patients with previously untreated locally advanced NSCLC. The results showed that vandetanib 100 mg/d in combination with either VC or GC was not tolerated and not a feasible first-line treatment for NSCLC [37]. A phase II randomized study was conducted to evaluate the efficacy and tolerability of vandetanib plus gemcitabine (V/G) compared with gemcitabine alone in advanced NSCLC. The explorative analysis showed that the median PFS was significantly prolonged in the P/G arm compared with the V/G arm (169 days VS 183 days, *P* = 0.047). The addition of vandetanib to gemcitabine was well tolerated [38].

#### Nintedanib

Nintedanib is a tyrosine kinase inhibitor of VEGFR, FGFR, and PDGFR, which was involved in angiogenesis. The phase I, open-label dose-escalation study investigated BIBF 1120 (Nintedanib) combined with PC in first-line patients with advanced IIIB/IVNSCLC. The data showed that 200 mg BIBF1120 combine with PC demonstrated an acceptable safety profile [39]. A phase II double-blind study to investigate the efficacy and safety of nintedanib, which failed first or second line platinum-based chemotherapy in advanced NSCLC. Although the median PFS (6.9 weeks) and OS (21.9 weeks) had no significant difference in nintedanib treatment, it was well tolerated and warrants further exploration [40].

### Combination of anti-angiogenic and chemotherapy in second-line of NSCLC (Table 3)

**Table 3:** Combination of antiangiogenic and chemotherapy in second-line of NSCLC

Agent	Target	Arms	No	Stage	ORR of (%)	mPFS	mOS	Note	Ref
Bevacizumab	VEGF	Bevacizumab+ different chemotherapy	40	Advanced non-squamous NSCLC	5.0	-	29.6	Had encouraging anti-tumor efficacy as second line therapy	[41]
		Bevacizumab+ Carboplatin/paclitaxel after EGFR mutations	31	IIIB/IV non-squamous NSCLC	37	6.6	18.2	Did not achieve the initial treatment goal	[42]
Nintedanib	VEGR PDGFR FGFR	Docetaxel+ placebo Docetaxel+ nintedanib	659 655	IIIB/IV NSCLC	-	2.7 3.4	9.1 10.1	Effective second-line combination with docetaxel	[43]
Sunitinib	VEGFR, PDGFR, FGFR cKIT	Pemetrexed Sunitinib Pemetrexed+Sunitin	42 47 41	IIIB/IV NSCLC	-	4.9 3.3 3.7	10.5 8.0 6.7	OS was significantly better with pemetrexed alone compared with the two sunitinib-containing arms	[44]
Vatalanib	VEGR PDGFR FGFR	1250 mg vatalanib once-daily Vatalanib 500 + 750 mg(2 divided dosing)	56 62	IIIB/IV NSCLC		2.1 2.8	7.3 9.0	Potential benefits in tumor size reduction and survival.	[46]
Ramucirumab	VEGF	Placebo-docetaxel Ramucirumab+docetaxel	81 76	NSCLC	18.5 28.9	4.21 5.22	14.65 15.15	Improved PFS with a manageable safety profile	[47]
		Placebo-docetaxel Ramucirumab+docetaxel	625 628	IV NSCLC	-	3.0 4.5	9.1 10.5	Improves survival as second-line treatment	[48]
Bavituximab	PS	Docetaxel + bavituximab 3 mg/kg Docetaxel + bavituximab 1 mg/kg	41 80	Advanced or metastatic NSCLC	17.1 13.8	4.5 3.3	11.7 7.3	High dose acquired longer PFS and os	[49]

#### Bevacizumab

It is well known that several clinical trials have authenticated bevacizumab with PC or GC and showed fantastic efficiency in the first-line therapy of advanced NSCLC. However, there is no study comparing the difference between first or second line therapy in patients with advanced NSCLC in China. The ORR were 23.1 % and 5.0 % in first and second line therapy (*P* = 0.020), respectively. The median OS were 27.2 months (95 % CI 13.3–41.1 months) and 29.6 months (95 % CI 6.7–52.5 months), respectively (*P* = 0.740). At last, the study concluded that the combination of bevacizumab and chemotherapy had encouraging anti-tumor efficacy as both first and second line therapy [41]. A phase II study to analyze the efficacy of bevacizumab plus PC in advanced NSCLC with EGFR mutated as first-line therapy. The ORR was 37% (90% CI; 24–52%), PFS was 6.6 months (95% CI; 4.8–12.0 months), and the median OS was 18.2 months (95% CI; 12.0–23.4 months). It is a pity that this research did not achieve the initial treatment goal [42].

#### Nintedanib

Several studies had tested the efficacy of nintedanib in the first-line of advanced NSCLC. The efficacy and safety of nintedanib in the setcond-line for NSCLC is still unclear. It is surprising that a large-scale clinical research LUME-Lung1 validated nintedanib in combination with docetaxel as an effective second-line option for patients with advanced NSCLC. A total of 655 patients were randomly assigned to receive docetaxel plus nintedanib and 659 patients received docetaxel plus placebo. The PFS was 2.7 months and 3.4 months in the docetaxel plus the placebo group and the docetaxel plus nintedanib group, respectively, indicating the patients benefit from the nintedanib group (*p* = 0.0019). The median OS had no significant change between the two groups; however, the OS was significantly improved for patients with adenocarcinoma histology in the docetaxel plus nintedanib group compared with the docetaxel plus placebo group (median 10.9 months vs 7.9 months, *p* = 0.0073). The LUME-Lung1 study demonstrated that nintedanib plus docetaxel is an effective second-line option for patients with advanced NSCLC who are resistant to the first-line treatment, especially for patients with adenocarcinoma [43].

#### Sunitinib

Sunitinib is a small-molecule inhibitor of multiple receptor tyrosine kinases, including VEGFR, PDGFR, FGFR1 and c-KIT. The CALGB 30704 study was conducted to validate whether sunitinib therapy improved outcomes in the second-line setting of advance NSCLC compared to chemotherapy. We divided 130 eligible patients into pemetrexed alone, sunitinib alone and pemetrexed plus sunitinib groups. The median PFS was 4.9 months (95% CI, 2.1– 8.8), 3.3 months (95% CI, 2.3–4.2), and 3.7 months (95% CI, 2.5–5.8) for pemetrexed alone, sunitinib alone and the pemetrexed plus sunitinib group, respectively (*p* = 0.18). The median OS was 10.5 months (95% CI, 8.3–20.2) for pemetrexed alone, 8.0 months (95% CI, 6.8–13.5) for sunitinib alone, and 6.7 months (95% CI, 4.1–10.1) for pemetrexed plus sunitinib (*p* = 0.03). In the subgroup analysis, there was no benefit in the PFS or OS of the pemetrexed plus sunitinib or sunitinib groups in either the squamous or non-squamous subsets. In conclusion, this study showed that the OS was significantly better with pemetrexed alone compared with the other group [44].

#### Vatalanib

Vatalanib (PTK787/ZK 222584, PTK/ZK) is an oral small molecular multi-tyrosine kinase inhibitor which prevents activation of VEGFR, PDGFR, and stem-cell factor receptor c-kit [45]. Patients with stage IIIB/IV NSCLC received a fixed dose of 1250 mg vatalanib either once-daily (QD) or twice daily (TDD: 500 mg a.m. + 750 mg p.m.) as second line treatment until disease progression or unacceptable toxicity. The PFS was 2.1 months for QD (95% CI, 1.0–2.9) and 2.8 for TDD (95% CI, 2.2–4.0). The OS was 7.3 months for QD (95% CI, 4.3–13.5) and 9.0 for TDD (95% CI,7.4–11.6) with a moderate toxicity profile. In a word, it seems that TDD acquired longer a PFS and OS compared to the QD of vatalanib. Nevertheless, these results need to be validated in further randomized trials [46].

#### Ramucirumab

Ramucirumab is a monoclonal antibody that specifically targets the domain of VEFR receptor 2. It is well known that ramucirumab improves the OS compared with an active comparator for previously treated NSCLC as the first new therapy [21]. On the other hand, a double-blind randomized placebo-controlled phase II study assessed the efficacy and safety of second-line ramucirumab-docetaxel in Japanese patients with NSCLC with disease progression after platinum-based therapy. The median PFS was longer with ramucirumab-docetaxel (5.22 months) than with placebo-docetaxel (4.21 months). The median OS was 15.15 months for placebo-docetaxel and 14.65 months for ramucirumab-docetaxel. The ORR was also longer in the ramucirumab-docetaxel arms. In conclusion, the Japanese patients with NSCLC acquired longer OS and PFS in the second-line of ramucirumab-docetaxel therapy [47]. A phase III trial named REVEL assessed the efficacy and safety of docetaxel plus ramucirumab or placebo as a second-line treatment for patients with stage IV NSCLC after platinum-based therapy resistance. The median OS was 10.5 months for 628 patients who received ramucirumab plus docetaxel and 9.1 months for 625 patients who received placebo plus docetaxel (HR 0.86, 95% CI 0.75−0.98; *p* = 0.023). The median PFS was 4.5 months for the patients in the ramucirumab group compared with 3.0 months for the control group (*p* < 0.0001). This large clinical study significantly proved that ramucirumab plus docetaxel improves survival as a second-line treatment of patients with stage IV NSCLC [48].

#### Bavituximab

In 2014, bavituximab was approved by the US Food and Drug Administration (FDA) as a potential treatment of second-line NSCLC. A randomized placebo-controlled phase II trial of docetaxel and bavituximab (1 and 3 mg/ kg) as a second-line treatment of NSCLC was conducted. Forty-one patients were treated with bavituximab at 3 mg/kg plus docetaxel, and the other 80 patients were treated with bavituximab at 1 mg/kg plus docetaxel. The ORR was 17.1% and 13.8% in the bavituximab at high dose plus docetaxel and the lower dose plus docetaxel, respectively. Regarding the secondary endpoints, the median PFS was 4.5 months and 3.3 months for 3 mg/kg bavituximab plus docetaxel and 1 mg/kg bavituximab plus docetaxel. The median OS was 11.7 months and 7.3 months for 3 mg/kg bavituximab plus docetaxel and 1 mg/kg bavituximab plus docetaxel, respectively. The safety of 3 mg/kg bavituximab plus docetaxel was similar to that of the control arm indicating the planned dose for the Phase III trial [49].

### Combination of antiangiogenic therapy and targeted therapy in NSCLC (Table 4)

**Table 4:** Combination of antiangiogenic therapy and targeted therapy in NSCLC

Agent	Target	Arms	No	Stage	ORR of (%)	mPFS	mOS	Note	Ref
Bevacizumab	VEGF	Erlotinib+bevacizumab Chemotherapy+ bevacizumab	63 61	Advanced non-squamous NSCLC	23.8 34.4	18.4ws 25.0ws	16.4 -	Did not show a benefit in terms of PFS for BE. OS was not reached in BC arms.	[51]
		Erlotinib+bevacizumab Cisplatin-gemcitabine+bevacizumab	111 113	Advanced non-squamous NSCLC	12 36	3.5 6.9	12.6 17.8	The ORR,PFS and OS were shorter in BE arm	[52]
		Gefitinib + bevacizumab	42	Advanced non-squamous NSCLC	73.8	14.4	-	OS had not yet been reached because of severe adverse events	[53]
		Erlotinib+ bevacizumab	109	IIIB/IV lung adenocarcinoma	-	13.2	-	Acquired benefit for the combined use of erlotinib and bevacizumab	[54]
Sunitinib	VEGFR, PDGFR, FGFR cKIT	Erlotinib Erlotinib + sunitinib	64 64	IIIB/IV NSCLC	3.0 4.6	2.0 2.8	7.6 8.2	The combination did not improve PFS	[55]
		Sunitinib+ pretreated EGFR-TKIs	30	pretreated EGFR-TKIs NSCLC	-	1.25	3.40	No sign of overall clinical benefits	[56]
Sorafenib	VEGFR PDGFR c-Kit	Sorafenib+ erlotinib Placebo+ erlotinib	111 55	IIIB or IV NSCLC	8 11	3.38 1.94	7.62 7.23	Did not statistically improve ORR or PFS combined with erlotinib	[59]
		Gemcitabine + sorafenib Erlotinib + sorafenib	31 29	Advanced NSCLC	6.5 10.3	-	6.55 12.6	Erlotinib plus sorafenib was feasible in elderly patients	[60]
		Sorafenib+ erlotinib	46	Advanced NSCLC	30.4	-	-	Well-tolerated and effective against advanced NSCLC	[61]

#### Bevacizumab

EGFR TKI, such as erlotinib and gefitinib, was the standard treatment for patients affected by NSCLC who harbored EGFR mutations. However, most patients are usually resistant to EGFR TKI treatment. Most of the trails had been under investigation to overcome the underlying mechanism of resistance to EGFR TKI, such as the MET amplification and EGFR T790M mutation [50]. The TASK study evaluated the efficacy and safety of the EGFR TKI erlotinib in combination with the bevacizumab as a first-line therapy in advanced non-squamous NSCLC patients. One hundred twenty-four patients were divided into two arms: bevacizumab plus chemotherapy (BC arm) or bevacizumab plus erlotinib (BE arm). The median PFS was 18.4 weeks versus 25.0 weeks for BE versus BC, respectively (*p* = 0.0183). The median OS was 16.4 months for BE and was not reached for BC (*p* = 0.4063). The ORR was also without significance between the two arms (23.8% with BE compared with 34.4% with BC, *P* = 0.19). In conclusion, this study did not show a PFS benefit for the BE combination in first-line advanced NSCLC compared with BC [51]. The INNOVATIONS study was designed to assess the efficacy of bevacizumab plus erlotinib in first-line patients with IIIB/IV NSCLC. The response rate was 12% VS 36% in bevacizumab plus erlotinib(BE arm) compared to the cisplatin, gemcitabine and bevacizumab(CGB) arm (*p* < 0.0001). Meanwhile, the ORR and OS were also longer in the CGB arm compared to the BE arm. This study indicated that the platinum-based combination chemotherapy remains the standard of care in first-line treatment of non-non-squamous NSCLC [52]. Another phase II study was conducted to assay whether bevacizumab enhances the effect of gefitinib in the EGFR mutant NSCLCs. Forty-two patients were enrolled in the study. The 1-year median PFS time were 14.4 months (95% CI 10.1–19.2). The median PFS differed significantly between the EGFR exon 19 deletion and the L858R point mutation (18.0 versus 9.4 months, respectively; p = 0.006). This study demonstrates gefitinib in combination with bevacizumab as the first-line therapy seems to be a favorable in EGFR mutated NSCLC [53]. An international multi-center single-arm phase 2 trial named BELIEF conducted done at 29 centers in eight European countries to evaluate the efficacy of erlotinib combined with bevacizumab in patients with EGFR-mutant NSCLC. The overall median PFS was 13.2 months (95% CI 10.3–15.5). In the T790M-positive group, the median PFS was 16.0 months (12.7 to not estimable) whereas in the T790M-negative group, the median PFS was 10.5 months. The BELIEF study provides strong evidence of the benefit for erlotinib combined with bevacizumab in patients with NSCLC who have EGFR mutations [54].

#### Sunitinib

A randomized double-blind multicenter phase II trial was designed to assess the efficacy and safety of sunitinib compared to erlotinib in patients with chemotherapy pretreated NSCLC. The median duration of the follow-up was 17.7 months. The median PFS was 2.0 versus 2.8 months for erlotinib alone versus sunitinib in combination with erlotinib (HR 0.898, *P* = 0.321). The OS was 7.6 versus 8.2 months (HR 1.066, *P* = 0.617), and the ORRs were 3.0% and 4.6%, respectively. However, it is disappointing to us that sunitinib combined to erlotinib did not significantly improve the PFS in patients with advanced NSCLC [55]. A trial was undertaken to evaluate the efficacy and potential toxicity of sunitinib therapy in advanced NSCLC patients pretreated with EGFR-TKIs in China. The PFS was 1.25 months (95% CI: 0.90–1.9 months), and the OS was 3.40 months (95% CI: 3.00–6.80 months). There is no sign of overall clinical benefits of sunitinib detected in patients with pretreated EGFR-TKIs in China. Whether sunitinib is beneficial or not for NSCLC patients pretreated with EGFR-TKIs requires further investigation [56].

#### Sorafenib

Sorafenib is an oral multi-kinase inhibitor targeting the receptor tyrosine, including receptors for VEGF and FDGFR and c-Kit. A multinational double-blind placebo-controlled monotherapy phase III trial named MISSION administered sorafenib as third-/fourth-line treatment with advanced NSCLC. The median OS was 8.2 months in the sorafenib (*n* = 350) and 8.3 months in the placebo groups (*n* = 353, *p* = 0.47). The median PFS was 2.8 versus 1.4 months (*p* < 0.0001). However, among 89 patients with EGFR mutations, the OS (13.9 versus 6.5 months; *p* = 0.002) and PFS (2.7 versus 1.4 months; *p* < 0.001) were significantly higher with sorafenib than the placebo. This study showed that, as third-/fourth-line treatment with advanced NSCLC, sorafenib did not significantly increase the OS, but did increase the PFS [57]. The CTONG 0805 study was designed to assay the efficacy and safety of sorafenib in patients with advanced lung adenocarcinoma after failure of EGFR-TKIs therapy in China. The median PFS and OS were 3.7 months [95% CI, 3.5–3.9 months] and 7.4 months (95% CI, 5.7–9.2 months), respectively. It showed that sorafenib monotherapy did not achieve positive results in patients in the CTONG 0805 trial [58]. A randomized double-blind placebo-controlled Phase II trial was designed to evaluate the efficacy of sorafenib plus erlotinib in advanced NSCLC. The median PFS was 3.38 months in the sorafenib-erlotinib group and 1.94 months in the erlotinib-placebo group (*P* = 0.196). The median OS was 7.62 months in the sorafenib-erlotinib group and 7.23 months in the placebo-erlotinib group (*P* = 0.290). In 67 patients with EGFR wild-type tumors, the median PFS was 3.38 months for sorafenib-erlotinib VS 1.77 months for the placebo- erlotinib group (*P* = 0.018). The median OS was 8 months for sorafenib-erlotinib VS 4.5 months for the placebo-erlotinib group (*P* = 0.019). The subset analyses in the EGFR WT showed a benefit for the combination of erlotinib-sorafenib; however, failed in all of the advanced NSCLC patients [59]. The aim of a multicenter, randomized phase II study was to evaluate the clinical activity and safety of sorafenib in combination with erlotinib or gemcitabine in unselected untreated elderly patients with NSCLC. The ORR was 6.5% for the gemcitabine plus sorafenib group and 10.3% for the combination of erlotinib plus sorafenib. The median OS was 6.55 months for the gemcitabine plus sorafenib patients and 12.6 months for the erlotinib plus sorafenib patients. The erlotinib-sorafenib combination was feasible in elderly patients with advanced NSCLC [60]. Recently, the KCSG-0806 study determined the clinical activity of sorafenib in combination with erlotinib in patients with advanced NSCLC. The overall response rate was 30.4%. In the EGFR mutation, the ORRs were 62.5%, 6.7% and 34.8%(*P* = 0.013) in the EGFR wild type and EGFR unknown tumor subgroup. It appears that sorafenib plus erlotinib is effective against advanced NSCLC [61].

### Anti-angiogenic combined with immunotherapy

#### The mechanism of anti-angiogenic regulated the tumor microenvironment

**Figure 2:**
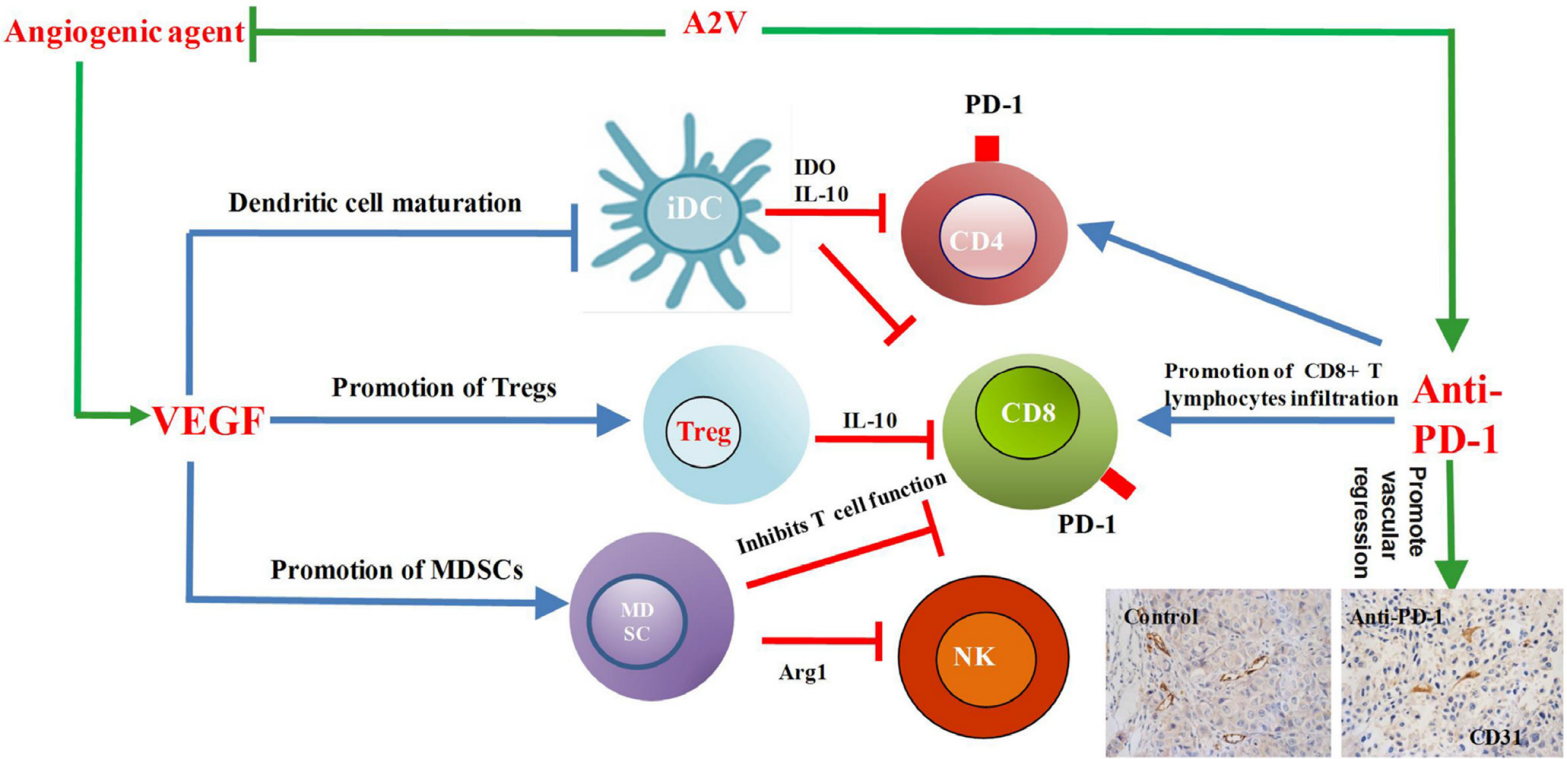
Combined inhibition of tumor angiogenesis and the immune checkpoint PD-1 regulated tumor microenvironment VEGF, vascular endothelial growth factor; iDC, immature dendritic cell; Treg, Tregulatory cell; MDSC, myeloid -derived suppressor cell; CD4, cluster of differentiation 4; CD8, cluster of differentiation 8; NK, natural killer cell; IDO, indoleamine 2; Arg1, arginase 1; IL-10, interleukin-10; PD-1, programmed cell death protein-1; A2V: bispecific antibody to angiopoietin-2 (ANGPT2) and Vascular endothelial growth factor A (VEGFA).

It is well known that the tumor microenvironment plays a key role in tumor progression. It is also obvious that anti-angiogenics can stimulate the immune system through altering the tumor microenvironment. Several studies showed that VEGF was an important factor in the immunosuppressive micro-environment that enable the tumor to evade immune-surveillance and induce angiogenesis through some mechanisms. It is reported that VEGF can promote and the induction of inhibitory immune-cell growth, including T-regulatory cells and myeloid derived suppressor cells (MDSCs), as well as inhibition of T-cell development (Figure 2). On the other hand, VEGF influences lymphocyte trafficking across the endothelia to the tumor by inhibiting lymphocyte adhesion. Because of defects in endothelial intercellular adhesion molecule-1 (ICAM-1) and vascular cell adhesion molecule-1 (VCAM-1), there is clustering at the endothelial cell surface, thereby blocking T-cell infiltration into tumors [62]. It is well known that anti-angiogenic agents can reduce tumor compactness and vasculature, resulting in improved oxygenation of the tumor microenvironment. In recent years, immunotherapies targeting the T-cell immune checkpoint receptor PD-1, or its ligand PD-L1 and cytotoxic T lymphocyte-associated protein 4 (CTLA-4), have led to significant improvements in some cancer prognosis.

#### Anti-angiogenic combined with PD-1 or CTLA-4 inhibitors in NSCLC

The recent success of programmed cell death- 1 (PD-1) and programmed cell death ligands (PD-L1 and PDL-2) in the treatment of cancer has emphasized the essential role of the eradication of tumors by preventing T-cell-mediated destruction. The PD-1 pathway is a T-cell inhibitory pathway that is induced by the binding of the PD-1 receptor on the T-cell plasma membrane to PD-L1 on the tumor. Tumor cells have blocked this pathway by up-regulating PD-L1 expression [63]. In the rapidly evolving area, PD-1 inhibitors nivolumab, pembrolizumab and atezolizumab are approved for the treatment of NSCLC. It is reported that sunitinib decreased PD-1 expression and increased the infiltration of CD-4+ T cells into the tumor [64]. Multiple trials are currently investigating combinations of anti-angiogenic agents and immunotherapies in NSCLC. The NCT01454102 trial studies the efficiency of bevacizumab plus nivolumab in the III/IV NSCLC. The median PFS was 37.1 weeks in the bevacizumab plus nivolumab arm and 21.4 weeks in the non-squamous patients in the nivolumab monotherapy arm. The ORR was almost the same between the two arms. However, the median OS was not reached in either arm. In addition, the bevacizumab plus nivolumab arm showed less toxicity. In conclusion, bevacizumab plus nivolumab improved the PFS for maintenance therapy in advanced NSCLC with a tolerable safety profile [65]. However, the clinical trials of combined treatment with the PD-L1 inhibitor ramucirumab plus pembrolizumab and bevacizumab plus atezolizumab in advanced non-squamous NSCLC are currently ongoing. For the treatment of anti-angiogenic agents combined with the CTLA-4 inhibitor in advanced NSCLC, there is no study to report to point us in a new direction in the future.

#### The application of anti-angiogenic agents combined with immunotherapy in *in vivo* tumor models to regulate the tumor microenvironment

Until now only two articles reported anti-angiogenic agents combined with immunotherapy for antitumor development in the NSCLC models. Endostatin is a 20-kDa fragment of type XVIII collagen that reducing the proliferation, invasion and migration of endothelial cells. It has been proved that rh- endostatin could improve patients’ PFS in advanced NSCLC with a combination of chemotherapy in clinical trials [66]. One of the studies at first indicated that rh-endostatin combined with the adoptive cytokine-induced killer cells (CIK cells) transferred to nude mice inhibited the growth of the lung carcinoma. The rh-endostatin could improve the tumor microenvironment by normalizing the tumor vasculature and reducing the hypoxic area. Furthermore, treatment with rh-endostatin significantly increased the homing of CIK cells and tumor-infiltration lymphocytes and decreased the accumulation of MDSCs in the tumor tissue *in vivo*, providing new insight for combining anti-angiogenesis therapy with immunotherapy in the treatment of NSCLC [67]. Furthermore, this team showed that bevacizumab augments CIK cells in the tumor and the combination of CIK cells significantly inhibits the growth of lung cancer in the mice models [68]. It is interesting to us that no reports have validated the role of anti-angiogenic combined with PD-1 and CTLA-4 inhibitors in the NSCLC models. One of the study showed sunitinib decreased PD-1 expression and increased the infiltration of CD-4^+^ T cells into the colorectal tumor. Combination of anti-PD-1 antibodies with VEGF-A inhibitors induced a strong antitumor role in colorectal tumor development [64]. Simultaneous blockade of VEGFR2 and PD-1 induced the anti-colon adenocarcinoma effect *in vivo*. Especially anti-VEGFR2, which inhibits tumor neovascularization and PD-1 blockade, enhanced the local immunity, including IFN-γ and TNF-α expression [69].The bispecific antibody (A2V), which blocks angiopoietin-2(ANGPT2) and VEGFA, provides anti-tumor benefits in metastatic breast cancer, pancreatic neuroendocrine tumors and melanoma, including the promotion of vascular regression, normalizing the remaining blood vessels and facilitating the perivascular accumulation of activated CD8+ cytotoxic T lymphocyte (CTL) infiltration. The novelty of this study is that it first identified A2V as an anti-tumor strategy that may unleash or increase the efficacy of anti-PD-1 for cancer immunotherapy [70] (Figure 2). Recently, a study found that the combination of anti-VEGFR2 and anti-PD-L1 antibodies induced the generation of endothelial venules(HEVs), which promoted lymphocyte infiltration through the activation of lymphotoxin β receptor (LTβR) signaling in breast cancer and pancreatic neuroendocrine cancers. Furthermore, anti-angiogenic therapy can improve anti-PD-L1 function that facilitates enhanced cytotoxic T cell (CTL) activity and tumor cell destruction [71]. A study investigated the effect of the combination of axitinib, a TKI against VEGFR-1, -2 and -3, with the therapeutic inhibition of CTLA-4 in subcutaneous and intracranial mouse melanoma models. The combination of axitinib with CTLA-4 inhibitor reduced tumor growth, increased survival and increased the number of CD4+ and CD8+ T cells, intratumoral DCs and suppressive MDSCs in both the intracranial and subcutaneous models [72].

## CONCLUSIONS

Taken together, brain metastases with advanced non-squamous NSCLC in patients who are more than 70 years old will acquire beneficial and improved PFS and OS with first-line for bevacizumab combined with chemotherapy [7, 13, 14]. However, one of the studies showed that the patients with NSCLC brain metastases seem likely to have extended PFS and OS compared to the non-brain metastases [10]. In addition, treatment of NSCLC brain metastases with bevacizumab in the first line requires further investigation. Second-line advanced non-squamous NSCLC patients are also suitable for accepting anti-angiogenesis therapy [41–49]. Anti-angiogenic agents combined with chemotherapy in the first-line or the second line or combine with EGFR-TKI target therapy in advanced NSCLC have shown significant improvements in the ORR, PFS or OS. Meanwhile, these studies did not show sufficient efficacy even though they used the same anti-angiogenic drug. This may be due to the preclinical data suggesting that anti-angiogenic therapies may enhance the stromal and microenvironment, eventually contributing to the different drug resistances and therefore limiting the benefit of these agents.

In recent years, combination immunotherapy with anti-angiogenic agents has become more popular in tumor treatment and have demonstrated potential benefits. However, which patients will acquire the most benefits from this novel therapy is still unknown. It is imperative for us to select biomarkers to aid in choosing these patients. Maybe this approach benefits patient with advanced NSCLC with poor prognosis, including the patients that did not acquire any benefit from checkpoint inhibitor monotherapy, or, for those who were PD-1 negative, failed in the first-line therapy. In addition to the patients’ selection, the most appropriate timing, best sequence and the dose of immunotherapy and anti-angiogenic agents is still a crucial problem. One study found that the high dose of anti-angiogenic agents combined with immunotherapy stimulated immune-activation [73].

In conclusion, it is important that more investigation be done to develop and clinically validate the anti-angiogenic agents combined with chemotherapy and TKI inhibitor treatments. The new findings indicate that the combination of anti-angiogenic agents and immune checkpoint blockades could have synergistic antitumor functions with less toxicity. However, more challenges remain to be overcome regarding whether combination treatments with chemotherapy, TKI inhibitors or immunotherapy can be realized.

## Abbreviations

FGF: Fibroblast growth factor
FGFR: Fibroblast growth factor receptors
EGF: Epidermal growth factor
EGFR: Epidermal growth factor receptor
VEGF: Vascular endothelial growth factor
VEGFR: Vascular endothelial growth factor receptor
PDGF: Platelet-derived growth factor
PDGFR: Platelet-derived growth factor receptor
VEGF: Vascular endothelial growth factor
iDC: Immature dendritic cell
Treg: T-regulatory cell
MDSC: Myeloid-derived suppressor cell
CD4: Cluster of differentiation 4
CD8: Cluster of differentiation 8
NK: Natural killer cell
IDO: Indoleamine 2
Arg1: Arginase 1
IL-10: Interleukin-10
PD-1: Programmed cell death protein-1
A2V: Bispecific antibody to angiopoietin-2 (ANGPT2) and Vascular endothelial growth factor A (VEGFA)
